# Population, sex and body size: determinants of behavioural variations and behavioural correlations among wild zebrafish *Danio rerio*

**DOI:** 10.1098/rsos.170978

**Published:** 2018-01-17

**Authors:** Tamal Roy, Anuradha Bhat

**Affiliations:** Department of Biological Sciences, Indian Institute of Science Education and Research (IISER) Kolkata, Mohanpur 741246, West Bengal, India

**Keywords:** predation, boldness–activity correlation, shoal-association, sex difference

## Abstract

This study (1) investigated variation among populations and the effects of sex and body size on boldness, activity and shoal-association tendency among wild zebrafish, and (2) tested for existence of correlations between behaviours, controlling for sex and body size. Individuals across four natural populations were tested for general activity in a novel situation, number of predator inspections undertaken and tendency to associate with a conspecific shoal in the presence of predators. Results showed a significant effect of population on boldness with a population from high-predation habitat being bolder than populations from low-predation habitats. Males showed significantly higher tendencies than females to associate with a conspecific shoal in the presence of predators. Further, a negative relationship was found between activity and boldness only within two low-predation populations. Individual body size had a strong effect on the activity–boldness relationship within the low-predation population from flowing water habitat. Smaller fish were bolder and less active while larger fish were more cautious and active. Overall, the results indicated that while population-level behavioural responses might be shaped by predation pressure, state-dependent factors could determine behavioural correlations among individuals within populations.

## 1. Introduction

Existence of behavioural correlations imply that plasticity in behavioural traits may be limited, thus constraining the ability of animals to behave in an optimal fashion across situations [1]. For example, when boldness and aggressiveness are positively correlated, boldness could be expressed as an outcome of selection for higher aggressiveness, even though boldness may not always be a favourable trait like in conditions of high predation [1]. For species with diverse geographical distributions, different behavioural types might be favoured in different ecological conditions and thus the existence of correlations would result in suboptimal behaviour in some environments [2]. This study investigated variation in behavioural traits and correlations between behavioural responses in different contexts [3] among four natural populations of zebrafish, *Danio rerio*. Here, a context refers to a functional behavioural category such as feeding, predator avoidance, predatory inspections or exploration [4].

The term ‘temperament’ has been used in various contexts by ethologists, and here we define it as an individual's response to a challenging situation, or behaviours indicating affect. Thus, ‘temperament trait’ is typically used with reference to a population. Individual differences in behaviour are often highlighted by temperament traits that can be broadly classified along five axes, namely shyness–boldness, exploration--avoidance, activity, aggressiveness and sociability [5]. Divergences in these behavioural traits have been demonstrated among populations of several fish species. For example, fishes from different predation regimes may show variable boldness–shyness responses depending on the measure and context [6–11]. While poeciliid *Brachyraphis episcopi* populations from high-predation regimes were more exploratory and active than low-predation populations [12], three-spined sticklebacks *Gasterosteus aculeatus* from high-predation habitats were less bold and active than those from low-predation habitats [8]. In our previous study, we found fish from high-predation habitats to be bolder than fish from low-predation habitats [13]. Recent studies investigating the role of different selective forces in determining temperament traits indicate that the direction of correlations between behaviours can actually vary across populations [1]. Existence of a strong relationship between aggression, activity and exploratory behaviour, for instance, was found in high-predation but not in low-predation pond three-spined sticklebacks [14]. By contrast, another study showed no evidence for linkage between aggression, boldness and feeding behaviours among marine and pond nine-spined stickleback *Pungitius pungitius* populations [15]. Moretz *et al*. [16] found significant differences between average levels of boldness and aggression across three laboratory strains of zebrafish but found no evidence for existence of a relationship between boldness and aggression within the three strains. Martins & Bhat [17], on the other hand, found a correlation between aggression and boldness only within a single population of zebrafish among the several populations that were studied. This indicated that the relationship between behaviours is shaped by forces acting at a population level rather than at individual level.

While ecological factors shape population-level responses, sex and body size can determine individual behavioural responses. Previous studies have demonstrated that males are significantly bolder than females [10,13,18,19]. Males and females have also been shown to differ in shoaling tendencies [20,21]. Body size influences risk-taking [13,22–24] and shoaling [25] in many species and this has been attributed to differential metabolic requirements . However, few studies have investigated the effects of sex and body size on relationships between behaviours. Larger individuals may be high explorers but more cautious to protect their larger reproductive assets (gonads) than smaller individuals [26]. Conversely, individuals with larger reproductive assets could be bolder and might take higher risks to gain more resources to maintain their assets [27]. Within fish populations, inter-individual differences in body size might be associated with sex differences where females are often larger than males. Therefore, relationships between behaviours might be expected to be different in populations where the males and females differ significantly in body size. Studies on sex differences indicate similar correlations between behavioural traits for males and females [28,29], although a study on wild passerines showed sex heterogeneity in relationships between behaviours [30]. A negative association between nestling defence and handling aggression was found in females whereas this association was positive and non-significant in male blue tits [30]. A recent study described the existence of a sex-specific relationship between boldness and shoaling in laboratory-reared zebrafish [31]. Our study investigated differences in boldness, activity and shoal-association tendency between the sexes and body size differences across four populations of zebrafish. Further, the variation in relationships between behaviours was investigated within populations, considering sex and body size of individuals.

Zebrafish occupy a range of habitats with diverse ecological conditions [13,32,33] and this makes the species suitable for comparative studies of behaviour. The goal of this study was twofold. First, the effects of population, sex and body size on the behaviours were investigated. Our previous study showed that fish from high-predation habitats were bolder and took greater risks to leave the association of shoal and feed in presence of predators [13]. We, therefore, hypothesized that individuals of populations from high-predation habitats would be bolder and more active and would be less likely to associate with a conspecific shoal than those from low-predation habitats. Further, smaller individuals demonstrate lower predator avoidance response than larger individuals [7]. Ingley *et al*. [18] showed that males of *Brachyrhaphis* fish are bolder than females. Our previous study showed that males were bolder than females and smaller individuals took greater risks to feed than larger individuals [13]. We, therefore, expected males to be more active, bolder and less inclined towards associating with a conspecific shoal than females. Simultaneously, we expected small fish to be bolder and more active than larger fish. Secondly, the existence of correlations between activity, boldness and shoal-association tendency within populations were tested based on one-time assessment of each behavioural trait, controlling for individual sex and body size. Bold individuals are often more active than shy individuals [34]. However, bold fish may shoal more than shy individuals as demonstrated by Way *et al*. [31] among zebrafish. We hypothesized that highly active individuals would be bold to leave the association of shoal and inspect predators.

## 2. Material and methods

### 2.1. Populations

We collected four populations of zebrafish from various locations in India—(1) Kalibazaar (KB, in Nadia district of West Bengal), a stagnant agricultural drain with silty substratum [13,35,36]; (2) Palakmati (PM, in Hoshangabad district of Madhya Pradesh) [13], a pool connected to a slow-flowing stream with small gravel and sandy substratum; (3) Kaushalya (KA, in Panchkula district of Haryana), a larger river with rocky substratum [13,33] and; (4) Asan (AS, in Dehradun district of Uttarakhand), a side-pool off a slow-flowing stream with silty–muddy substratum [13,33] (table 1). Collections were made using cast nets and drag nets during the pre-monsoon season (March–April) of 2013. The piscivorous fish species co-occurring in the habitats with zebrafish were identified and their numbers recorded to estimate the total relative abundance of predatory fishes (table 1). The predatory fishes found included *Channa punctatus* and *Macrognathus pancalus* in the site Kalibazaar, and *Channa orientalis, Amblyceps mangois* and *Mastacembelus armatus* in the site Kaushalya [13]. No piscivorous fish were recorded in the sites Palakmati and Asan during sampling [13].

**Table 1.** Environmental and ecological variables of the four water bodies.

site	altitude (m)	water velocity (m s^−1^)	total relative abundance of predatory fishes
Asan (AS)	514.20	0	0
Palakmati (PM)	312.11	0.66	0
Kalibazaar (KB)	19.0	0	0.01
Kaushalya (KA)	406.60	0.32	0.04

### 2.2. Rearing and maintenance

We collected approximately 150 fishes from each site and transported in oxygenated bags to the laboratory. Populations were maintained separately. Batches of 30 individuals (mixed sex) were housed together in bare housing glass aquaria (45.7 × 25.4 × 25.4 cm) with a standard corner filter. This rearing density was uniform for all populations. Holding room temperature was maintained at 25°C and lighting was set at 14 : 10 h light : dark cycle to mimic natural conditions. The fish were fed freeze-dried blood-worms and *Artemia* alternately, once daily in the morning. The fishes were maintained for eight months before the commencement of experiments to ensure full grown adults.

We used live predators, Snakeheads (*Channa* spp.), known to occur commonly in zebrafish habitats across India and feed on zebrafish and other small fishes [13], for the experiments to quantify boldness (predator inspection) and shoal-association tendency (described in the section below). We captured six snakeheads (approx. 12 cm body length) using cast nets from local water bodies and transferred in oxygenated bags to the laboratory. The snakeheads were kept in three bare glass aquaria similar to the experimental tanks (61 × 30.5 × 30.5 cm), fed with standard pellet food and were acclimated to the laboratory conditions (holding room temperature 25°C, 14 : 10 h light : dark cycle) for at least a month before being used in the experiments.

### 2.3. Experiments

Three kinds of assays were performed on individuals across populations in order to test boldness (predator inspection), activity and shoal-association tendency—(1) predator inspection assay for testing boldness [37], (2) activity assay [38] and (3) assay for shoal-association tendency [31]. Sixty individuals were randomly selected from a population at a time. Thus, a total of 240 individuals were assayed. Each individual was tested across the three assays but only once for a particular assay. To minimize stress and possible habituation to the experimental set-up, we conducted assays on 60 fishes over 2 days followed by an intermission of 2 days before commencement of the next assay. The order of the behavioural assays across individuals within and between populations was randomized. The experiments on each population lasted for 10 days. The four populations were, therefore, assayed over a period of 2 months. The sex and the total length of all individuals were recorded before commencement of experiments.

Individual fishes were kept in separately labelled 1 l cylindrical plastic containers (filled with 520 ml water) throughout the period of experiments, i.e. for 10 days. This method of isolation was essential to allow tracking of individual behaviour over the course of experiments [13,32,33,35,36] as each individual was tested across three assays. Light : dark conditions, temperature and feeding of individuals in the containers were maintained similar to those in the housing tanks. All experiments commenced during the same time of the day and fishes were fed in their respective containers only after the end of the assays for that day.

#### 2.3.1. Predator inspection assay

**Figure 1.**
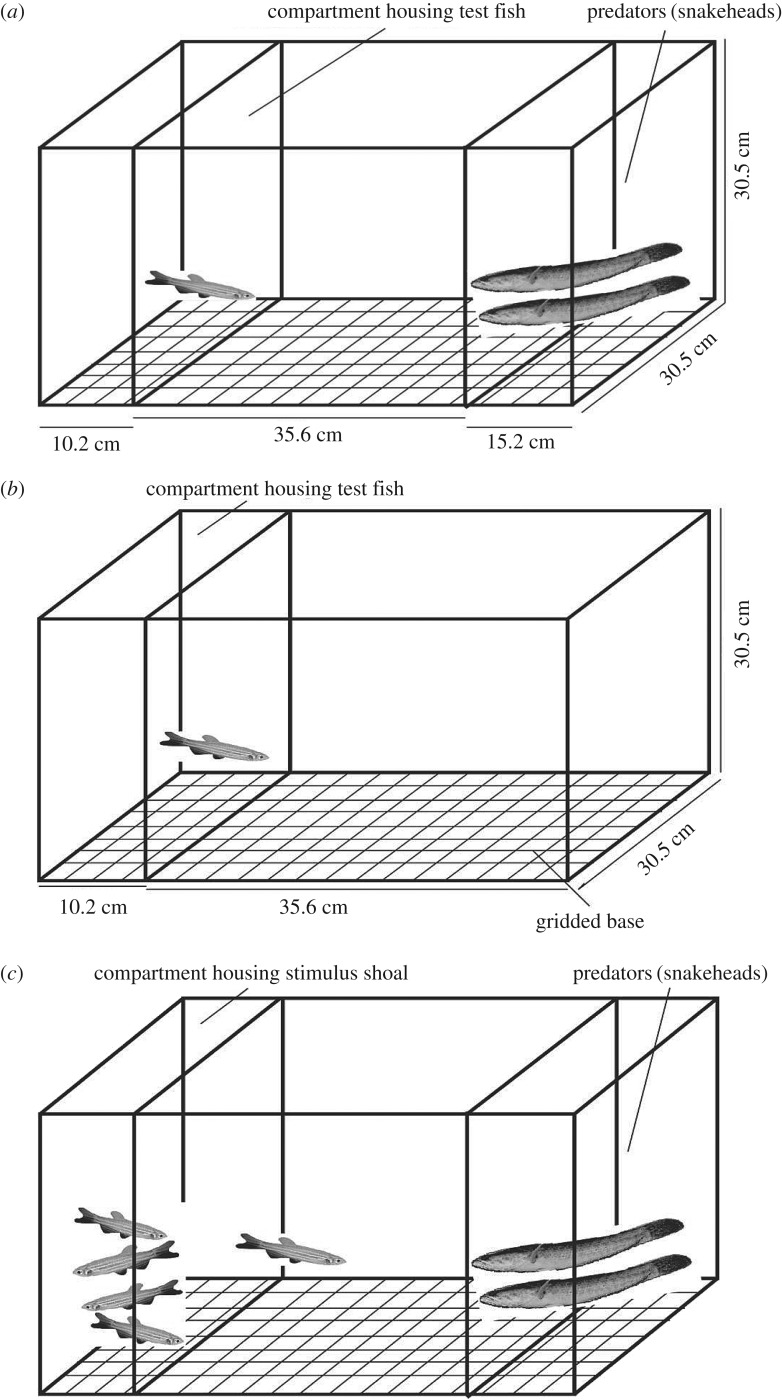
Diagrams showing experimental set-up (side view) for assays (*a*) predator inspection, (*b*) general activity of fish and (*c*) tendency to associate with conspecific shoal in presence of predators.

We used a bare glass aquarium (61 × 30.5 × 30.5 cm) for the experiments. The aquarium was divided into three compartments with removable barriers of fibreglass (figure 1*a*). An end compartment (10.2 × 30.5 × 30.5 cm) with a removable opaque partition was kept for placing the test fish, the middle compartment (35.6 × 30.5 × 30.5 cm) was the testing arena and the other end compartment (15.2 × 30.5 × 30.5 cm), with a transparent perforated fibreglass screen (to allow for visual and chemical cues), was kept for the two predatory fishes. The sides of the experimental tank were lined with brown cardboard paper to avoid disturbances during the assays.

The night prior to running the experiments, the experimental tank was filled with aerated water to a depth of 12.7 cm. Two snakeheads were released into the predator compartment and allowed to settle overnight. The assay was carried out the following day. A test fish was gently transferred to the test fish compartment (figure 1*a*) and allowed to acclimate for 2 min. After that, the opaque fibreglass partition between the test fish compartment and the rest of the arena was removed. The test fish could then see the predators through the transparent perforated screen but could not pass through to their compartment. The behaviour of the fish was filmed for 10 min using a HD camcorder Canon LEGRIA HF R306 overlooking the set-up. From the video recordings, the number of inspections undertaken by the test fish during the 10 min period was noted. We defined an inspection as the fish being within one body length of the predator compartment.

#### 2.3.2. Activity assay

A bare glass aquarium (45.8 × 30.5 × 30.5 cm) was used for the experiments, the base of which was marked with 4 × 4 cm grids. The arena was divided into two compartments with removable barriers of fibreglass. A narrow compartment with an opaque partition (10.2 × 30.5 × 30.5 cm) housed the test fish initially and the broad compartment (35.6 × 30.5 × 30.5 cm) was the testing arena (figure 1*b*).

The tank was filled with aerated water to a depth of 12.7 cm. A test fish was gently transferred to the narrow compartment (figure 1*b*) and allowed to acclimate for 2 min. The opaque partition separating the test fish compartment and the rest of the arena was then removed. The fish behaviour was filmed for 10 min using a HD camcorder. From the video recordings, we noted the number of grids a test fish crossed every 1 min after the first 5 min of the recording. The number of grids traversed per min was taken as the measure of activity.

#### 2.3.3. Tendency to associate with shoal in presence of predators

We used a similar set-up as for the predator inspection assay. One of the side compartments (10.2 × 30.5 × 30.5 cm) had two partitions in place separating the rest of the arena, a fixed transparent perforated fibreglass window screen followed by an opaque removable one. A group of four zebrafish of the same population as that of the test fish, randomly selected from the stock tanks, was placed in this compartment an hour before the commencement of experiments to act as a stimulus shoal (figure 1*c*). The broad middle compartment was the testing arena and the other side compartment held two predatory fishes.

Two snakeheads were released into the predator compartment and allowed to settle overnight. The following day the test of tendency to associate with the conspecific shoal in presence of predators was conducted. A test fish was gently transferred to an opaque cylindrical release chamber kept close to the compartment with stimulus shoal (figure 1*c*) and allowed to acclimate for 2 min following which the cylindrical release chamber and the opaque removable partition before the fixed screen separating the shoal chamber and testing arena was slowly lifted. Once released, the test fish could see the predators as well as the stimulus shoal through the transparent window screens but could not pass through to their compartments. The behaviour of the test fish was filmed for 10 min using the HD Canon camcorder. From the video recordings, we recorded the total time an individual spent within one body-length distance of the conspecific shoal (association) in presence of predators during the 10-min period. To minimize observer bias during population studies, blinded methods were used when all behavioural data were recorded and analysed.

### 2.4. Statistical analysis

All data analyses were conducted using StatistiXL v. 1.8 software and R software ‘ppcor' package [39]. A multivariate analysis of covariance (MANCOVA) was conducted with individual measures of boldness (number of predator inspections), activity (number of grids traversed per min) and shoal-association tendency (time spent in association with conspecific shoal) as dependent variables, ‘population', ‘sex' and the interaction population × sex as factors and ‘Body size' as covariate. This was followed by univariate tests, analyses of covariance (ANCOVA), for effects of population, sex and body size separately for each behaviour. Post hoc comparisons (Mann–Whitney *U* tests) were conducted on paired sets of populations (with Bonferroni corrections for multiple comparisons) to compare differences in behaviour.

To test the existence of behavioural correlations, a multivariate Pearson correlation analysis was conducted within each population between individual measures of boldness, activity and shoal-association tendency. Also, a partial correlation analysis (using the Pearson method) between the behavioural measures was conducted within each population, controlling for sex and body size. The results of the partial correlation were compared with the results of the multivariate correlation to check for the effect of sex and body size on the relationships between behaviours. As the partial correlation method controlled for the effect of sex and body size, if there was any difference in the relationship found in the two methods/approaches, this would indicate that the variation in either of the behaviours is influenced by sex and/or body size.

## 3. Results

**Figure 2.**
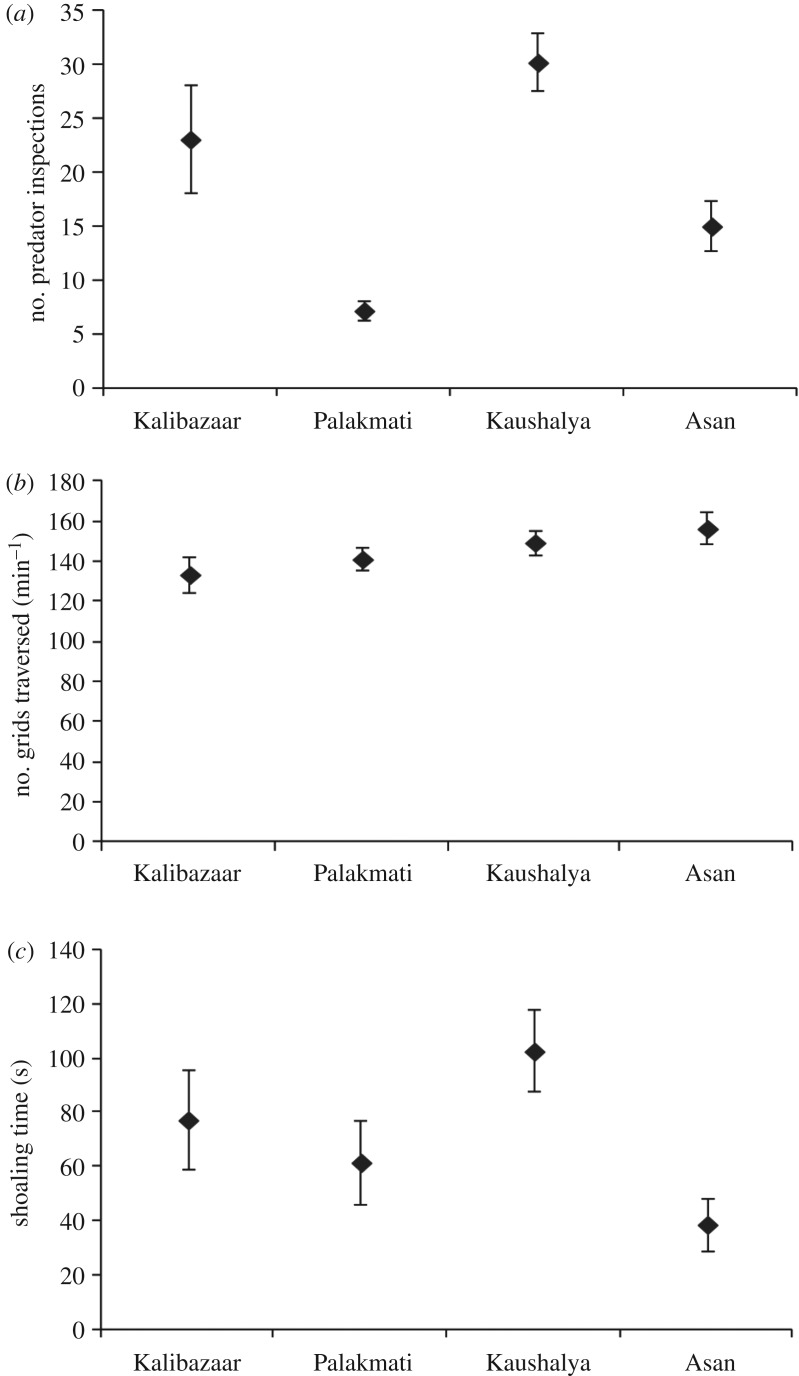
Comparisons of (*a*) predator inspection (boldness), (*b*) activity and (*c*) shoal-association time across populations. The mean ± s.e. values are (*a*) KB: 23.1 ± 4.95, PM: 7.25 ± 0.98, KA: 30.22 ± 2.71 & AS: 15.07 ± 2.32 (predator inspection), (*b*) KB: 133.6 ± 9, PM: 141.3 ± 5.77, KA: 149.53 ± 5.81 and AS: 156.53 ± 7.89 (activity), and (*c*) KB: 77.35 ± 18.35 s, PM: 61.67 ± 15.6 s, KA: 102.72 ± 15.19 s and AS: 38.78 ± 9.64 s (shoal-association time).

Overall, the multivariate analysis showed a significant effect of population (*F*_9,557 _= 3.67, *p *< 0.001) but no significant effects of sex (*F*_3,229 _= 1.63, *p* = 0.18) or body size (*F*_3,229 _= 1.36, *p* = 0.26) on behaviours. The results of univariate tests showed a significant effect of population only on boldness (*F*_3,231 _= 6.85, *p *< 0.001) and a significant effect of sex only on shoal-association tendency (*F*_1,231 _= 4.37, *p* = 0.038) (table 2). Boldness was found to be highest for high-predation KA population and the least for low-predation PM population (KA > KB > AS > PM) (figure 2*a*). Post hoc tests revealed significant differences in boldness between populations KB and PM (Mann–Whitney test, *U* = 2369.5, *p* = 0.003), KB and KA (Mann–Whitney test, *U* = 2429, *p* = 0.001), PM and KA (Mann–Whitney test, *U* = 3127, *p *< 0.001) and KA and AS (Mann–Whitney test, *U* = 2676, *p* < 0.001) (figure 2*a*). Thus KA differed significantly in boldness from all other populations (figure 2*a*). Also, males (83.33 ± 11.08 s) spent significantly more time in association with conspecific shoal in presence of predators than females (54.53 ± 10.08 s) (Mann–Whitney test, *U* = 8310, *p* = 0.03).

**Table 2.** Tests of univariate effects, analysis of covariance (ANCOVA) to test effect of population, sex and their interaction on each behavioural trait. Significant results are shown in bold (*p* < 0.05).

*Y* variable	source	*F*	*p*
activity	body size	1.22	0.27
	population	1.94	0.12
	sex	0.07	0.79
	population × sex	1.15	0.33
predator inspection	body size	1.47	0.22
	population	6.85	**<0.001**
	sex	0.13	0.72
	population × sex	1.02	0.39
shoaling tendency	body size	0.91	0.34
	population	2.23	0.08
	sex	4.37	**0**.**03**
	population × sex	0.12	0.95

**Figure 3.**
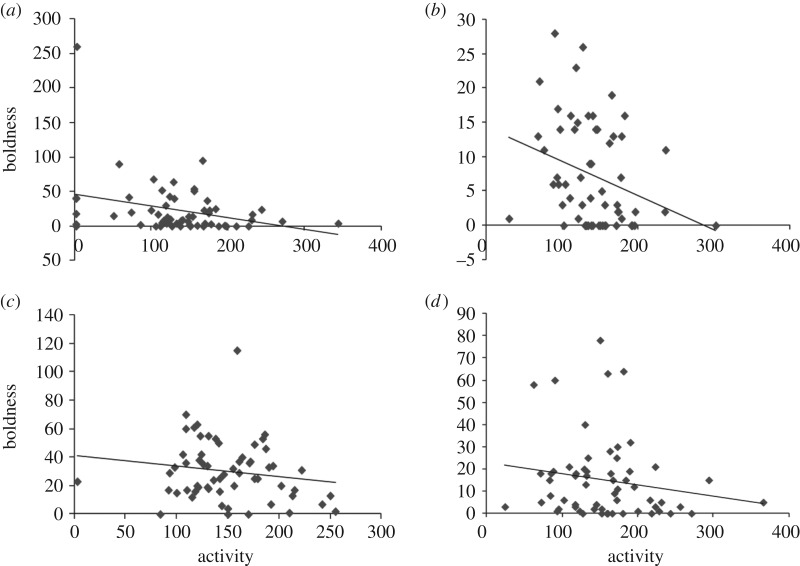
Scatter plots showing correlations between activity (no. of grids traversed per min) and boldness (no. of predator inspections undertaken) for populations (*a*) Kalibazaar (KB, *r* = −0.31), (*b*) Palakmati (PM, *r* = −0.29), (*c*) Kaushalya (KA, *r* = −0.16) and (*d*) Asan (AS, *r* = −0.17). Only populations KB and PM showed a significant relationship between activity and boldness.

A significant negative correlation was found between boldness and activity within KB (*n* = 60, *r* = −0.31, *p* = 0.016) and PM (*n* = 60, *r* = −0.29, *p* = 0.02) (table 3 and figure 3). However, when controlled for sex and body size, a negative correlation was found only within population KB (*n* = 60, *r* = −0.3, *p* = 0.02) (table 3), indicating that variation in either of the two behaviours (boldness or activity) could be explained by sex and/ or body size. A Pearson's correlation conducted between activity and boldness measures separately for PM males and females did not show significant results (males: *n* = 31, *r* = −0.28, *p* = 0.12; females: *n* = 29, *r* = −0.21, *p* = 0.27). Though non-significant, the estimates of correlation between activity and boldness were negative for both sexes. However, a significant negative relationship was found between body size and boldness (*n* = 60, *r* = −0.31, *p* = 0.02) while a significant positive relation between body size and activity (*n* = 60, *r* = 0.37, *p* = 0.004) for individuals of PM.

**Table 3.** Pearson correlation results for the temperament traits. The first part shows the results of Pearson's correlation between activity, boldness and sociability. The second part shows the results from the partial correlation between the three behaviours where the Pearson product–moment correlation coefficient has been adjusted to take into account the control variables, sex and body size. Significant results are shown in bold (*p* < 0.05).

pairs of behaviours	Kalibazaar	Palakmati	Kaushalya	Asan
correlation matrix (R)				
activity × boldness	**−0.31**	**−0**.**29**	−0.16	−0.17
activity × sociability	0.14	−0.17	0.22	0.05
boldness × sociability	0.09	0.04	−0.04	0.08
partial correlation matrix (*r*)				
activity × boldness	**−0**.**30**	−0.21	−0.16	−0.21
activity × sociability	0.17	−0.16	0.23	0.06
boldness × sociability	0.09	0.06	−0.01	0.13

## 4. Discussion

### 4.1. Variations in behaviour among populations—effects of predation and sex

The results showed a significant population effect only for boldness, which was measured by frequency of predator inspections undertaken by an individual (figure 2*a*). Among the different ecological factors, predation is one of the most important factors that shape behaviour [9,11]. The frequency of predator inspections (which is taken as the measure of boldness in the study) could be possibly related to the extent of predation pressure in the natural habitat [40]. Individuals of KA showed highest predator inspection tendencies while individuals of PM and AS made fewer predator inspections. KB fish made fewer inspections than KA but more than PM and AS. KA fish belonged to a habitat with high predation pressure while KB fish belonged to a low-predation habitat (table 1). No piscivorous fish were recorded from the PM and AS habitats (table 1). These results appear to indicate that fish from high-predation habitats are likely to be bolder than fish from low-predation habitats. Further studies across more populations differing in predation would be needed to investigate its role as a clear determinant of boldness. Our results are in agreement with a previous study on guppies where fish from high-predation stream habitats spent more time inspecting predators than fish from low-predation habitats [41]. Another study on minnows showed that fish from a predator-sympatric population inspected a potential predator more frequently than fish from predator-allopatric population [42]. This is in contrast to the expected notion that predator-sympatric populations need to be more cautious in occasions of predation hazard than predator-allopatric populations [43,44]. Individuals in predation pressure conditions still need to forage and reproduce in the face of predation risk. Thus, inspecting a potential predator would be necessary for assessing risks associated with daily activities. Such a selection force could be lacking in predation-allopatric habitats where zebrafish could be naturally aversive toward inspecting novel predators. Further studies with more populations would help understand the relationship between predation pressure and risk-taking tendencies among zebrafish. Populations from varying levels of predation pressure could be tested under controlled conditions to assess differences in behavioural responses between them. The role of predation on factors such as boldness is often intertwined with other ecological factors such as habitat complexity and food availability. Thus further experiments in controlled mesocosms could help in disentangling the role of each of these factors in influencing behaviour among wild populations.

While increased activity allows greater feeding opportunities, it also increases predation risk. Thus, activity responses across populations from different predation risk environments were expected to be different. The present study, however, did not reveal population differences in activity levels of zebrafish (figure 2*b*). Furthermore, individual sex or body size did not influence activity patterns in general in zebrafish. Again, while there were no population differences in shoal-association tendency (figure 2*c*), males were generally found to show a higher tendency to associate with conspecific shoal than females in presence of predators. This contradicts the expectation that males would show lesser shoal-association tendency than females. Shoaling behaviour in fish functions primarily as a defence against predators [44]. Females, which generally have greater reproductive investment than males, would be expected to shoal more strongly in order to avoid predation [40,44]. However, shoaling decisions in zebrafish could be governed by the sex of the fish in the shoal and males can identify females in the stimulus shoal based on visual cues [45]. Shoaling behaviour in males appears to be influenced by sexual selection pressures and males would tend to associate more with shoals having females than just males [45]. Therefore, it is likely that in our study, there were females among individuals of stimulus shoal selected randomly from the population stock, which could have influenced male shoaling tendencies.

### 4.2. Variations in activity–boldness relationship among populations—role of sex and body size

A significant relationship between activity and boldness was observed only within populations KB and PM (figure 3). Differential within-population behavioural correlations have been reported in a recent study on wild zebrafish for aggression–boldness [18]. In three-spined sticklebacks, existence of consistent correlations between activity, boldness and aggression within some populations but not in others have been attributed to differing levels of predation [6,14,46]. Juvenile and adult sticklebacks that were more active were also bolder and more aggressive only within one population [46]. Under natural conditions, the extent of individual activity levels would represent a trade-off between higher foraging rates and higher risks of predation [47]. Individuals that are generally active in the absence of predators (and thus feed at high rates) may also take inappropriate risks in the presence of predators [48]. A negative relationship was, however, observed between activity and predator inspection (boldness) within populations KB and PM that originated from habitats with low predation pressures. A study by Jones & Godin [38] showed that individuals who were more active in a novel environment reacted to a predator later than the less active individuals. Their results were consistent with the economic hypothesis according to which more exploratory fish would devote more attention to foraging rather than predator vigilance [49]. In the present study, as the chances of encountering predators in the native environments was low for individuals of KB and PM populations, higher activity among fish may translate to greater engagement in finding food resources than inspecting potential predators. While predation is a predominant selective force shaping correlated evolution of traits, [14] other factors such as intraspecific competition (for food) could also play a significant role [50]. Thus, abundance of food could drive increased activity levels towards foraging, while being less bold towards inspecting potential predators. Further studies are required to disentangle the role of these factors in shaping the relationships between traits in wild populations.

The results of the partial correlation analysis suggest that while sex and body size of individuals had no effect on the relationship between activity and boldness within population KB, there is a possible influence of body size on activity–boldness correlation within population PM. Smaller fish in PM showed greater number of predator inspections but were less active whereas larger fish were more active but initiated fewer predator inspections. The propensity to take risks (boldness) has been shown to be correlated with body size in fishes. Differences in behavioural responses between small and large fish could be a consequence of different physiological constraints [51]. Studies by Krause *et al*. [51] on sticklebacks suggests that smaller fish are subject to both higher predation and starvation risks than large ones, which could be because weight loss in smaller individuals has a greater proportional effect. They speculate that larger fish can also afford to be ‘extra' cautious because of the low cost of lost feeding opportunities, compared to smaller fish which have higher energy demands [51]. Another theory that might explain this observation is the ‘*Asset protection principle*' which states that the larger the accumulated (reproductive) assets, the more important it becomes to protect it [26]. Hence, the lower risk-taking by the larger fish could be to protect their greater accumulated reproductive assets than smaller conspecifics [22]. Exploring novel (potential) foraging grounds poses relatively lower threat than inspecting predators. Therefore, larger individuals could turn into more active explorers than the smaller ones [26] probably in order to be able to find food.

Sex of individuals did not affect correlations between behaviours. Sex differences in behavioural types can result from differential life-histories between sexes [52] and one can expect sex differences in consistent correlations between multiple traits, as has been reflected in wild passerines [30], comb-footed spiders *Anelosimus studiosus* [28] and water striders [53]. There are very few studies on sex differences in behavioural correlations among fish. A similar correlation structure for aggression-activity syndrome was found in males and females among reef fish sharpnose sandperch *Parapercis cylindrica* [29]. It is possible that such sex differences could be specific for mating related and social behaviours and may not show up across behavioural correlations comprising of only social behaviours.

In conclusion, while populations did not differ strongly in terms of activity and shoal-association tendency, they differed significantly in boldness. High-predation fish were bolder than low-predation fish. Tendency to associate with conspecific shoal is influenced by sex of the individuals and male zebrafish tend to associate with a shoal more than the females. Bolder individuals were less active than their more cautious counterparts. Again, within a low-predation population, smaller individuals were bolder but less active compared to the larger ones. Further experiments testing for consistent correlations between boldness and activity across multiple contexts are warranted to understand the occurrence of syndromes and tease out the relative roles of predation and other biotic factors in wild populations. Repeated measures on individuals on trait responses within contexts would also help understand within-individual variations on behaviours as well as their correlations. Multivariate (bivariate) models can be used to estimate among-individual correlation between behaviours [54]. Thus, these studies would then help in determining the specific role of factors such as body size variations in behaviours such as boldness and/or activity.

## Supplementary Material

Individual responses for the three behavioural assays.

## Supplementary Material

READ.TXT

## Data accessibility

All data for this study are provided in the electronic supplementary material, File1_Roy&Bhat.xls.
